# Risk of hospitalization with neurodegenerative disease after moderate-to-severe traumatic brain injury in the working-age population: A retrospective cohort study using the Finnish national health registries

**DOI:** 10.1371/journal.pmed.1002316

**Published:** 2017-07-05

**Authors:** Rahul Raj, Jaakko Kaprio, Miikka Korja, Era D. Mikkonen, Pekka Jousilahti, Jari Siironen

**Affiliations:** 1 Department of Neurosurgery, University of Helsinki and Helsinki University Hospital, Helsinki, Finland; 2 Department of Public Health, University of Helsinki, Helsinki, Finland; 3 Institute for Molecular Medicine Finland (FIMM), Helsinki, Finland; 4 Department of Anesthesiology, Intensive Care, Emergency Care and Pain Medicine, University of Helsinki and Helsinki University Hospital, Helsinki, Finland; 5 National Institute for Health and Welfare, Helsinki, Finland; Barts and the London School of Medicine & Dentistry Queen Mary University of London, UNITED KINGDOM

## Abstract

**Background:**

Previous epidemiological studies suggest that working-aged persons with a history of moderate-to-severe traumatic brain injury (TBI) may have an increased risk for developing neurodegenerative disease (NDD) while persons with a history of mild TBI do not. In this comprehensive nationwide study in Finland, we assessed the risk of NDD and history of moderate-to-severe TBI in the working-age population.

**Methods and findings:**

We performed a population-based follow-up study using the Finnish Care Register for Health Care to identify all persons between the ages of 18 and 65 years hospitalized during 1987–2014 due to TBI who did not have a baseline NDD diagnosis. We compared the risk of hospitalization with NDD between persons hospitalized due to moderate-to-severe TBI (intracranial lesions) and persons hospitalized due to mild TBI (no intracranial lesions). Follow-up NDD diagnoses were recorded from 1 year following the TBI to the end of 2014. NDD diagnoses included dementia, Parkinson disease, and amyotrophic lateral sclerosis. We used a Cox proportional hazards model, adjusting for age, sex, education, and socioeconomic group, to assess the association between TBI and NDD. In total, 19,936 and 20,703 persons with a history of moderate-to-severe TBI and mild TBI, respectively, were included. The overall time at risk was 453,079 person-years (median 10 years per person). In total, 3.5% (*N* = 696) persons in the moderate-to-severe TBI group developed NDD compared to 1.6% (*N* = 326) in the mild TBI group. After adjusting for covariates, moderate-to-severe TBI was associated with an increased risk for NDD, with a hazard ratio (HR) of 1.8 (95% CI 1.6–2.1) compared to mild TBI. Of the NDD subtypes, only moderate-to-severe TBI was associated with an increased risk for dementia (HR 1.9, 95% CI 1.6–2.2). Yet, this large-scale epidemiological study does not prove that there is a causal relationship between moderate-to-severe TBI and NDD. Further, the Care Register for Health Care includes only hospitalized persons; thus, patients diagnosed with NDD in the outpatient setting may have been missed. Additional limitations include the potential for miscoding and unmeasured confounds.

**Conclusions:**

In working-aged persons, a history of moderate-to-severe TBI is associated with an increased risk for future dementia but not for Parkinson disease or amyotrophic lateral sclerosis.

## Abstract 2

In a retrospective cohort study using the Finnish national health registries, Rahul Raj and colleagues investigate the association between traumatic brain injury and dementia, Parkinson disease, and amyotrophic lateral sclerosis.

## Author summary

**Why was this study done?:**

- Traumatic brain injury is a leading cause of death and disability worldwide, especially among young adults.
- Previous studies on the association between traumatic brain injury and neurodegenerative diseases have controversial findings. Furthermore, association studies with a long follow-up time are scarce.

**What did the researchers do and find?:**

- We conducted a retrospective, register-based nationwide study, identifying 19,936 persons (18–65 years) with a history of moderate-to-severe traumatic brain injury and 20,703 persons (18–65 years) with a history of mild traumatic brain injury admitted to hospitals between 1987 and 2014 in Finland.
- After adjusting for confounding factors, we found that persons with moderate-to-severe traumatic brain injury had a 90% increased probability of developing dementia compared to persons with mild traumatic brain injury.
- The risk was increased only for dementia, not for Parkinson disease or amyotrophic lateral sclerosis.

**What do these findings mean?:**

- Moderate-to-severe traumatic brain injury seems to be associated with a risk of future dementia in young and middle-aged adults.
- Further studies are required to examine causal factors contributing to the found association.

## Introduction

Traumatic brain injury (TBI) is a globally increasing healthcare problem, affecting persons of all ages [1]. Following the early phases of TBI, patients face a significant risk of long-term disability and neurological morbidity [2]. Previous epidemiological studies have found an association between history of TBI and risk for future neurodegenerative disease (NDD) (a concept including dementia, Parkinson disease [PD], and amyotrophic lateral sclerosis [ALS]), but the results have been conflicting and few studies have focused on the working-age population [3–7]. Gardner et al. showed that persons under 65 years with a history of mild TBI did not have an increased risk for dementia compared to non-TBI controls [8]. The development of NDD is supposedly most deleterious in the working-age population, as this not only causes significant morbidity but also has major socioeconomic consequences. Yet, to our knowledge, no previous studies have specifically looked at the overall risk for developing NDD in working-aged persons hospitalized due to TBI.

Our aim is to contrast the risk of NDD in working-aged persons hospitalized due to moderate-to-severe TBI to that of persons hospitalized due to mild TBI. Persons with a history of mild TBI and moderate-to-severe TBI are similar in regard to TBI-specific risk factors, such as alcohol use, which is why individuals with mild TBI serve as a suitable control group [9]. Further, since data on the possible effects of socioeconomic factors on the association between TBI and NDD are lacking, we adjust for education and socioeconomic group [10]. We hypothesized that working-aged persons with a history of moderate-to-severe TBI would have an increased risk for future NDD compared to persons with a history of mild TBI, after adjusting for covariates.

## Methods

### Ethical statement

The National Institute for Health and Welfare (THL/1326/5.05.00/2015) approved of the study, in accordance with Finnish national legislation. Statistics Finland (Dnro TK-53-1179-16) and the Population Register Centre (Dnro 1873/410/16) granted us access to their databases. The Finnish Office of the Data Protection Ombudsman (Dnro 2794/402/2015) approved the data collection and combining of data registries. The study was conducted according to the Declaration of Helsinki of the World Medical Association.

### Study design and setting

We used the nationwide Finnish Care Register for Health Care to identify persons treated in a public hospital due to TBI in Finland during 1987–2014. The Care Register for Health Care (a continuation of the previous Hospital Discharge Register) was established by the National Institute for Health and Welfare and contains data on persons discharged from every public hospital in Finland from 1969 onwards. The Finnish healthcare system is tax-funded by local municipalities and by the state. In practice, acute care of TBIs is provided solely by public nonprofit healthcare providers and not by private institutions. Thus, the Care Register for Health Care comprehensively includes persons hospitalized due to TBI. The same register can also be used to identify persons hospitalized with NDD. Previous studies have verified the diagnostic accuracy of the registers [11,12].

### Study population definition and exposure assessment

The study population consisted of working-aged persons (18–65 years) hospitalized due to moderate-to-severe TBI or mild TBI between 1 January 1987 and 31 December 2014. Mild TBI was defined as a discharge diagnosis indicating no traumatic intracranial lesion (ICD-9 850; ICD-10 S06.0) according to US Centers for Disease Control and Prevention (CDC) criteria, with the exception of isolated skull fractures [13]. Moderate-to-severe TBI was defined as a discharge diagnosis indicating traumatic intracranial lesion (ICD-9 851–854; ICD-10 S06.1–S06.9).

To diminish the likelihood of persons in the mild TBI group having significant traumatic intracranial lesions, we included only those hospitalized for less than 1 day. To diminish the likelihood of persons in the moderate-to-severe TBI group having no significant traumatic intracranial lesion, we included only persons hospitalized for 3 days or longer. In the case of several hospitalizations due to moderate-to-severe TBI or mild TBI, we used the first date of the most severe head injury (i.e., if the person first had a mild TBI and later a moderate-to-severe TBI, the latter was used).

### Follow-up and outcome assessment

**Fig 1:**
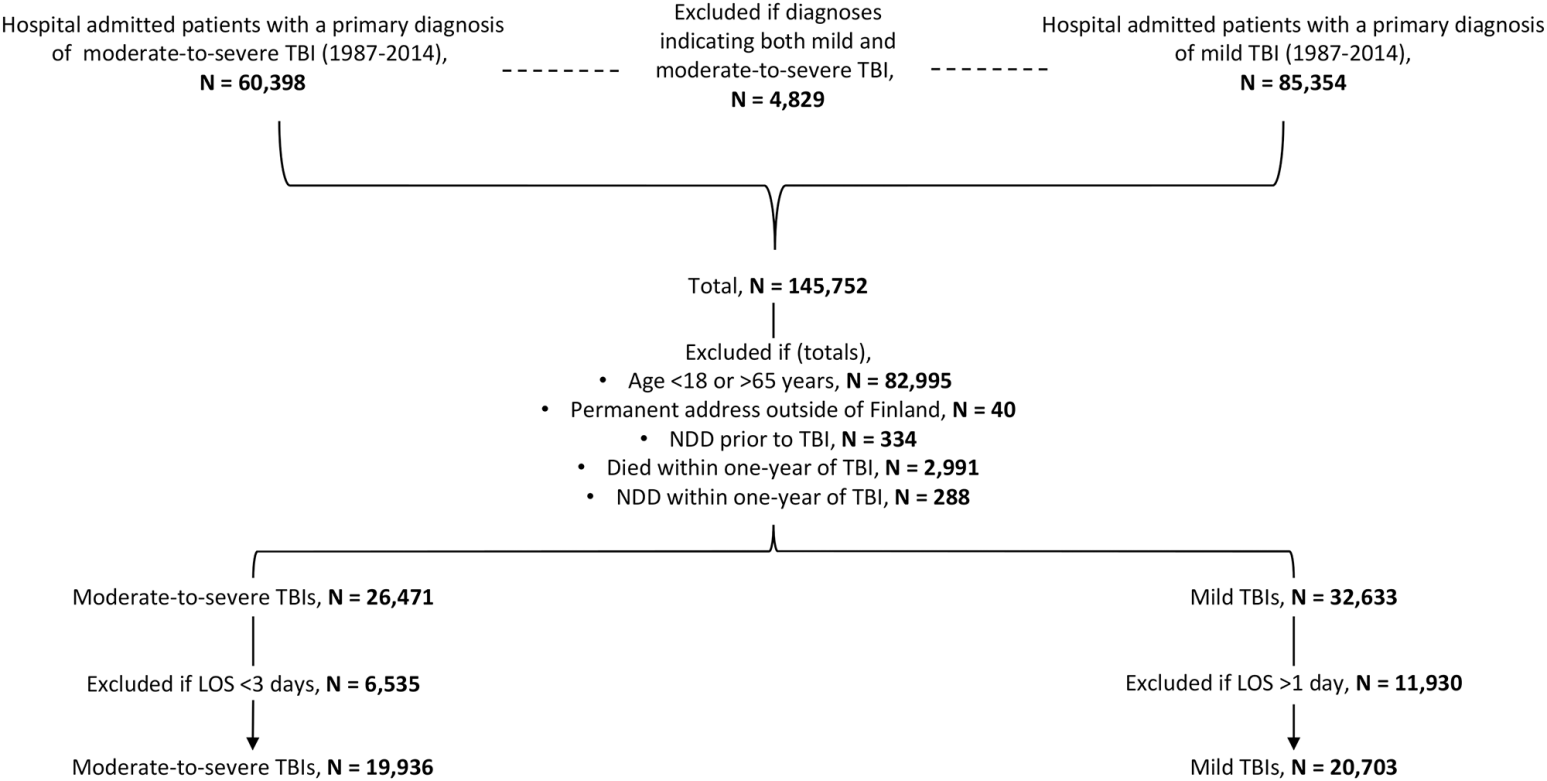
Flow chart showing study population. Out of the 288 persons excluded due to NDD diagnosis within the first year after the TBI, 203 belonged to the moderate-to-severe TBI group, and 85 to the mild TBI group. LOS, length of stay in hospital; NDD, neurodegenerative disease; TBI, traumatic brain injury.

The study population was prospectively followed up from time of TBI until diagnosis of NDD, death, emigration, or end of follow-up on 31 December 2014. We identified persons hospitalized, for any reason, who were diagnosed with a new NDD diagnosis of dementia (ICD-9 290, 331, 797; ICD-10 G30, F00, F01, F02, F03), PD (ICD-9 332; ICD-10 G20), or ALS (ICD-9 335.2; ICD-10 G12.2) from the Care Register for Health Care. The admission date for the hospitalization including the new NDD diagnosis was defined as the date of diagnosis. We excluded persons with a NDD diagnosis prior to the TBI and persons permanently living outside of Finland. We further excluded persons who received a NDD diagnosis or died within 1 year of the TBI to diminish the possibility of reverse causality. Fig 1 shows a flow chart of the selection and follow-up protocol.

### Definition of covariates

Statistics Finland classification of socioeconomic groups is based on the United Nation’s recommendations for the 1990 population censuses. The education classification system is based upon the International Standard Classification of Education 1997 and 2011 classifications. Data on socioeconomic group and highest level of education were obtained for the year closest to the end of follow-up. The classification systems are presented in S1 Text.

Data on mortality and date of emigration were obtained from the Population Register Centre of Finland. Information on date of birth, sex, hospitalization dates, and diagnoses came from the Care Register for Health Care. Data on socioeconomic group and education were obtained from Statistics Finland. We used the unique identification number assigned to all Finnish citizens to identify individuals and combine data from the registers.

### Statistical analysis

Descriptive characteristics of the cohort are presented either as categorical data (*N* [percent] and compared using a two-sided χ^2^ test) or as continuous data (mean [standard deviation] and compared using a *t* test). We calculated the unadjusted rates of NDD per 100,000 person-years. This was done overall and in prespecified age groups, given the increase in risk of NDD with age. Data are presented as NDD rate per 100,000 person-years with 95% confidence intervals.

We used Cox proportional hazards models in Stata (version 14, StataCorp, College Station, TX) to estimate covariate-adjusted hazard ratios (HRs) with 95% confidence intervals. Date of NDD was set as date of study exit. Persons dying before the end of follow-up or moving outside of Finland before the end of follow-up were censored at the time of death or emigration. Date of study entry was the hospital admission date for the hospitalization due to TBI. Age (continuous variable) was the underlying time parameter in all analyses. Socioeconomic group and level of education were treated as categorical variables, using the group with the most individuals as the reference category.

In the primary analysis, we used NDD as a composite outcome variable. In the sensitivity analyses, we separately assessed the risk for dementia, PD, and ALS. Subgroup analyses were conducted by sex, by prespecified age groups (18 to 40 years, 41 to 50 years, 51 to 60 years, and 61 to 65 years), and by equally sized hospital length of stay quartiles (3–5 days, 6–10 days, 11–24 days, ≥25 days). Hospital length of stay served as a surrogate marker of TBI severity [14].

We repeated the analysis using the Cox proportional hazards model in a matched sample within the cohort. We matched persons with a history of moderate-to-severe TBI and mild TBI in a 1:1 fashion based on age, sex, education, and socioeconomic group using the “ccmatch” function in Stata. If there were multiple mild TBI cases that matched a moderate-to-severe TBI case with respect to these variables, the “ccmatch” function included all of them.

The results are presented as HRs with 95% confidence intervals. We used the group of persons with a history of mild TBI as the reference group in all analyses. *p*-Values < 0.05 were considered statistically significant. We derived log–log plots of survival curves of TBI to verify that the proportional hazards assumption was not violated.

The reporting of this study is in accordance to the REporting of studies Conducted using Observational Routinely-collected health Data (RECORD) statement (S1 RECORD Checklist) [15].

The preplanned statistical plan is presented in S2 Text. One additional analysis assessing how the risk of dementia behaves as a function of time (in the matched cohort) when including persons diagnosed with dementia within the first year after TBI was performed in response to a reviewer comment.

## Results

### Baseline characteristics

A total of 19,936 persons with a history of moderate-to-severe TBI and 20,703 persons with a history of mild TBI were identified (Fig 1). Baseline characteristics of the two groups are shown in Table 1. Persons in the moderate-to-severe TBI group were on average 7 years older at the time of injury than persons in the mild TBI group (46 versus 39 years). The male to female ratio was higher in the moderate-to-severe TBI group than in the mild TBI group, although men predominated in both groups. There were no major differences in educational level between the groups. Nearly half of all persons had an upper-secondary level of education, while the higher education levels contained between 4% and 9% of individuals. Twenty-nine percent of persons in the moderate-to-severe TBI group died during follow-up, compared to 12% in the mild TBI group.

**Table 1:** Baseline characteristics of persons hospitalized due to moderate-to-severe and mild traumatic brain injury.

Characteristic	All persons (*N* = 40,639)	
	Moderate-to-severe TBI (*N* = 19,936)	Mild TBI (*N* = 20,703)
**Age at injury**		
Mean (SD), years	46 (13)	39 (15)
18–40 years	6,319 (31%)	10,722 (51%)
41–50 years	4,725 (24%)	4,112 (20%)
51–60 years	5,931 (30%)	4,043 (20%)
61–65 years	2,961 (15%)	1,826 (9%)
**Sex**		
Male	15,419 (77%)	12,711 (61%)
Female	4,517 (23%)	7,992 (39%)
**Socioeconomic group**		
Self-employed	538 (3%)	1,244 (6%)
Upper-level employees	611 (3%)	1,437 (7%)
Lower-level employees	1,052 (5%)	2,742 (13%)
Manual workers	1,711 (9%)	3,435 (17%)
Students	295 (1%)	691 (3%)
Pensioners	13,059 (66%)	7,657 (37%)
Unemployed	1,835 (9%)	2,598 (13%)
Unknown	835 (4%)	899 (4%)
**Education level**		
Upper-secondary	8,137 (41%)	9,603 (46%)
Short-cycle tertiary	1,708 (9%)	1,783 (9%)
Bachelor or equivalent	931 (4%)	1,456 (7%)
Master or equivalent	747 (4%)	1,068 (5%)
Doctor or equivalent	89 (1%)	111 (1%)
Unknown	8,324 (41%)	6,682 (32%)
Hospital length of stay*		
3–5 days	4,859 (24%)	NA
6–10 days	5,060 (25%)	NA
11–24 days	5,103 (26%)	NA
≥25 days	4,914 (25%)	NA
**NDD type**	696 (3.5%)	326 (1.6%)
Dementia	615 (3.1%)	276 (1.3%)
PD	68 (0.3%)	41 (0.2%)
ALS	13 (0.1%)	9 (0.1%)
**Mean age at NDD**	67 (19)	71 (10)
≤65 years	275 (1.4%)	84 (0.4%)
>65 years	421 (2.1%)	242 (1.2%)
**Time to NDD, years**		
Mean (SD)	10 (6)	14 (7)
Median (IQR)	9 (5–15)	14 (8–19)
**Censored persons**		
Died during follow-up prior to NDD^†^	5,739 (29%)	2,395 (12%)
Emigrated during follow-up prior to NDD^‡^	81 (0%)	153 (1%)

All differences between the two groups are statistically highly significant (p < 0.001).

*Hospital length of stay due to the TBI. All persons in the mild TBI group had a length of stay of 0 to 1 day.

^†^Does not include persons dying within 1 year of the TBI. These persons have already been excluded.

^‡^Does not include persons living permanently outside of Finland at the time of the TBI. These persons have already been excluded.

ALS, amyotrophic lateral sclerosis; IQR, interquartile range; NA, not applicable; NDD, neurodegenerative disease; SD, standard deviation; TBI, traumatic brain injury; PD, Parkinson disease.

### Unadjusted risk for neurodegenerative disease

Overall time at risk was 453,079 person-years (mean 11 years [SD 8], median 10 years [IQR 4–17], per person). During the follow-up, 696 (3.5%) persons with a history of moderate-to-severe TBI developed NDD compared to 326 (1.6%) of those with a history of mild TBI (*p <* 0.001). Dementia was the most frequent NDD, followed by PD and ALS, in both groups. Persons in the moderate-to-severe TBI group were on average 4 years younger than persons in the mild TBI group at the time of NDD diagnosis (67 versus 71 years). Significantly more persons in the moderate-to-severe TBI group were diagnosed with NDD before the age of 65 years compared to the mild TBI group (40% versus 26% of all NDD cases in the respective groups, *p <* 0.001). Persons with a history of moderate-to-severe TBI who went on to develop NDD tended to have a longer hospital length of stay than those who did not develop NDD, suggesting higher TBI severity.

There were no significant differences between persons in the moderate-to-severe TBI group and persons in the mild TBI group who developed NDD in terms of age, socioeconomic group, or level of education (S1 Table). The male to female ratio was higher among individuals in the moderate-to-severe TBI group who developed NDD than among individuals in the mild TBI group who developed NDD.

The unadjusted rate of NDD was 331 per 100,000 person-years in the moderate-to-severe TBI group (318 per 100,000 in men and 373 per 100,000 in women) and 134 per 100,000 person-years in the mild-TBI group (115 per 100,000 in men and 162 per 100,000 in women). The unadjusted rates for all three NDD subtypes were notably higher in the moderate-to-severe TBI group than in the mild TBI group (Table 2). The incidence of NDD increased with age. In the two youngest age groups (18–40 and 41–50 years), the rate of NDD was three to five times higher in persons with moderate-to-severe TBI compared to mild TBI, whereas in the two older age groups (51–60 and 61–65 years), the incidence was approximately one and a half times higher in the moderate-to-severe TBI group.

**Table 2:** Sensitivity and subgroup analyses for incidence and hazard ratios of neurodegenerative disease by sex and age group in persons with moderate-to-severe traumatic brain injury compared to persons with mild traumatic brain injury at baseline.

Subgroup	Unadjusted incidence per 100,000 person-years (95% CI)		HR (95% CI)	*p*-Value
	Moderate-to-severe TBI	Mild TBI		
**NDD type**				
Dementia	293 (270–317)	114 (101–128)	1.9 (1.6–2.2)	<0.001
PD	32 (26–41)	17 (12–23)	1.3 (0.9–1.9)	0.22
ALS	6 (4–11)	4 (2–7)	1.3 (0.5–3.2)	0.53
**Risk for NDD by sex**				
Female	373 (323–430)	162 (138–189)	1.9 (1.5–2.4)	<0.001
Male	318 (291–347)	115 (99–134)	1.7 (1.4–2.1)	<0.001
Risk for NDD by age group*				
18–40 years	43 (31–60)	9 (5–16)	1.8 (0.9–3.5)	0.08
41–50 years	178 (145–218)	62 (44–89)	2.7 (1.8–4.2)	<0.001
51–60 years	589 (527–659)	340 (288–401)	2.0 (1.6–2.4)	<0.001
61–65 years	1,152 (1,020–1,301)	891 (756–1,050)	1.4 (1.1–1.7)	0.003
**Risk for NDD by hospital LOS**				
0–1 days (mild TBI)	NA	134 (120–150)	Reference	NA
3–5 days^†^	241 (201–289)	NA	1.3 (1.1–1.6)	0.009
6–10 days^†^	332 (287–383)	NA	1.8 (1.5–2.1)	<0.001
11–24 days^†^	382 (334–438)	NA	2.0 (1.6–2.3)	<0.001
≥25 days^†^	362 (313–418)	NA	2.1 (1.7–2.5)	<0.001

A HR significantly over 1.0 indicates an increased risk for the outcome (NDD or NDD subtype). All HR analyses are adjusted for age, sex, level of education, and socioeconomic group, except for the male and female subgroup analyses, where only age, socioeconomic group, and level of education were adjusted for.

*Age at the time of head injury.

^†^Moderate-to-severe TBI.

ALS, amyotrophic lateral sclerosis; CI, confidence interval; HR, hazard ratio; LOS, length of stay; NA, not applicable; NDD, neurodegenerative disease; PD, Parkinson disease; TBI, traumatic brain injury.

### Adjusted risk of neurodegenerative disease

**Fig 2:**
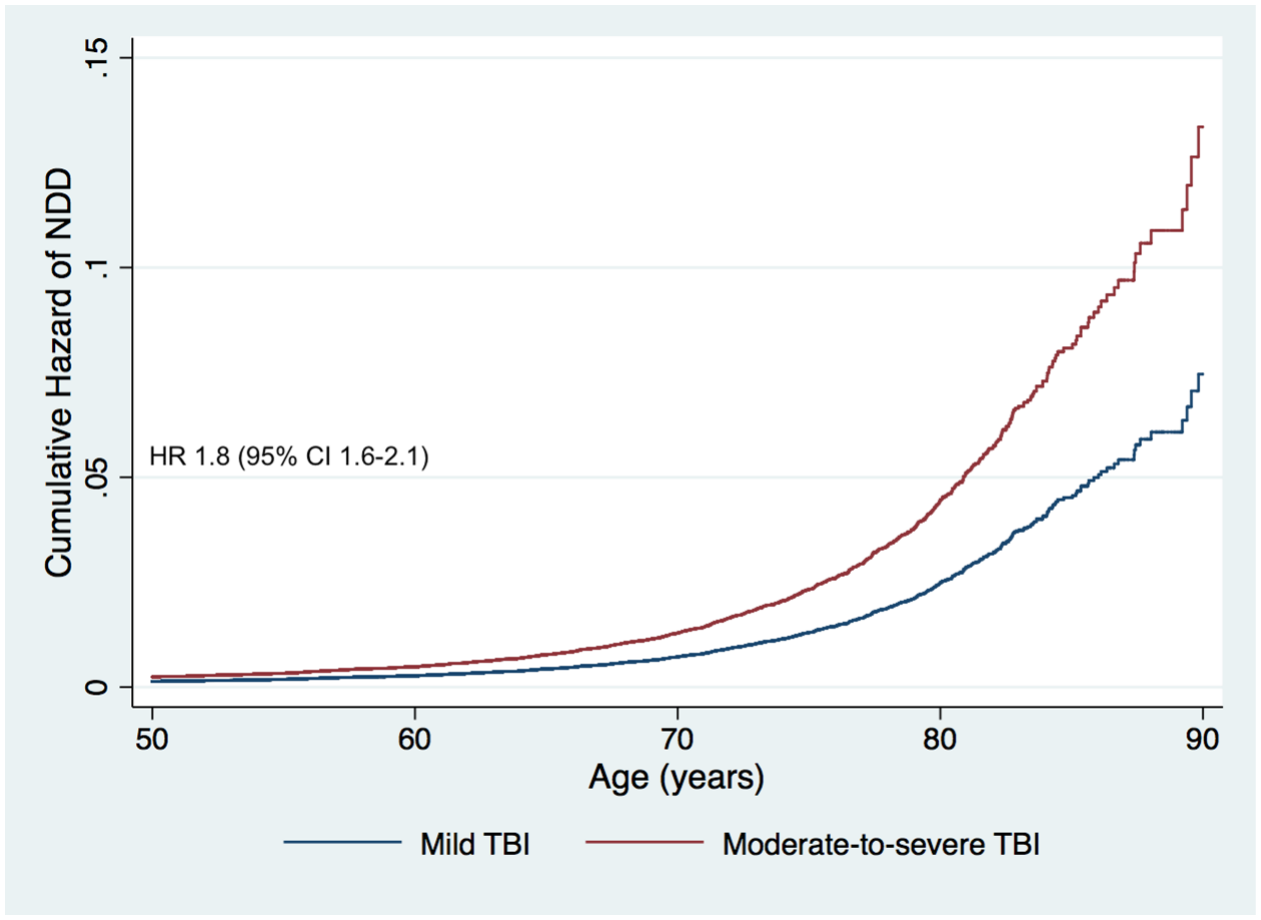
Cumulative hazard risk comparison for persons with a history of moderate-to-severe traumatic brain injury versus mild traumatic brain injury (adjusted for age, sex, level of education, and socioeconomic group). The HR shows the increased risk of NDD in persons with a history of moderate-to-severe TBI compared to those with a history of mild TBI. HR, hazard ratio; NDD, neurodegenerative disease; TBI, traumatic brain injury.

In the primary analysis, adjusting for age, sex, level of education, and socioeconomic group, moderate-to-severe TBI was associated with an increased risk of NDD, with a HR of 1.8 (95% CI 1.6–2.1, *p <* 0.001) compared to mild TBI (Fig 2). When analyzing all persons, female sex was associated with a decreased risk of NDD (HR 0.8, 95% CI 0.7–0.9, *p <* 0.001).

**Fig 3:**
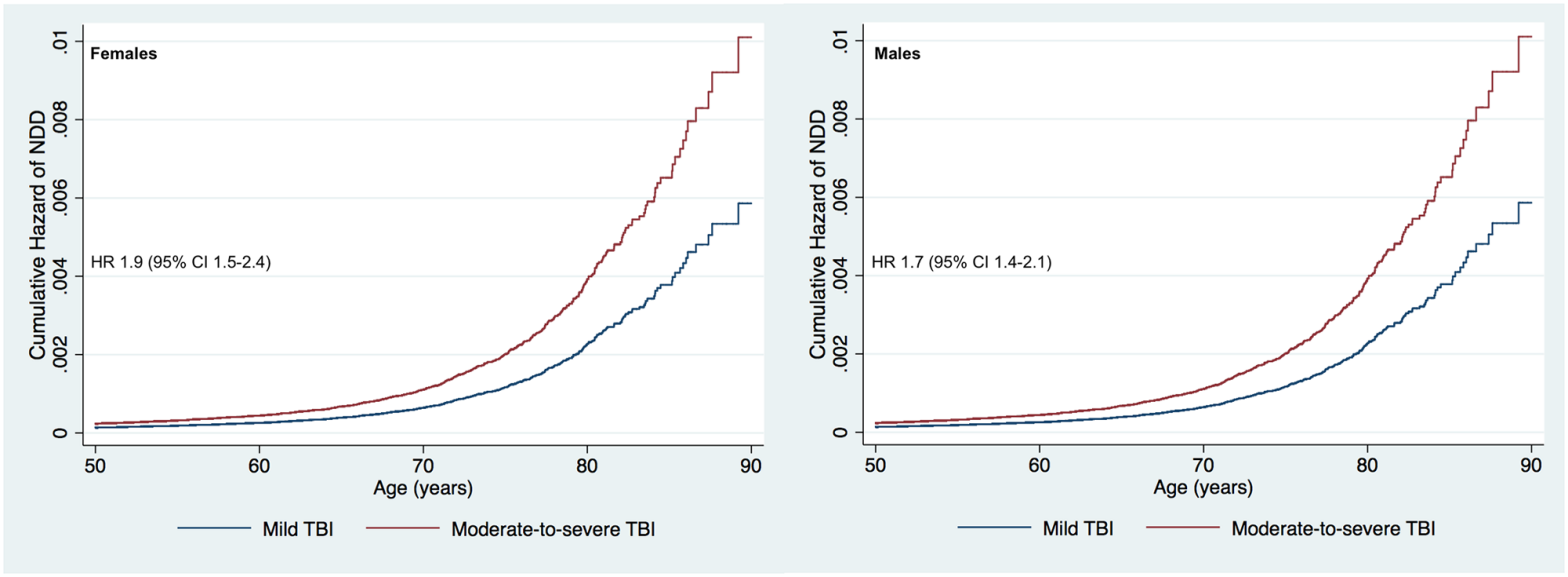
Cumulative hazard risk comparison for persons with a history of moderate-to-severe traumatic brain injury versus mild traumatic brain injury (adjusted for age, sex, level of education, and socioeconomic group). Women shown to the left, and men to the right. The HR shows the increased risk of NDD in persons with a history of moderate-to-severe TBI compared to those with a history of mild TBI for both females and males. HR, hazard ratio; NDD, neurodegenerative disease; TBI, traumatic brain injury.

The sensitivity (for NDD subtypes) and subgroup analyses (by sex, age group, and hospital length of stay) are shown in Table 2. In the sensitivity analyses, moderate-to-severe TBI was associated with an increased risk for dementia compared to mild TBI (HR 1.9) but not for PD or ALS. When analyzing women separately, moderate-to-severe TBI was associated with an increased risk for NDD compared to mild TBI, with a HR of 1.9. When analyzing men separately, moderate-to-severe TBI was associated with an increased risk for NDD with a HR of 1.7 (Fig 3). The relative risk of NDD among those with a history of moderate-to-severe TBI was highest among those aged 41 to 50 years and 51 to 60 years of age at baseline (HR 2.7 and 2.0, respectively), though the differences between age groups were not significant (overlapping 95% CIs). Increasing TBI severity, as reflected by duration of hospitalization, was associated with an increased risk for NDD (HR 1.3 to 2.1) in a dose–response pattern.

### Matched sample

We identified a total of 25,747 exposure–control matched persons within the follow-up cohort, of which 13,470 were persons with a history of mild TBI, and 12,277 persons with a history of moderate-to-severe TBI. There were no major differences in age, sex, level of education, or socioeconomic group distribution between the matched groups (S2 Table). In the matched sample, 3.2% of persons with a history of moderate-to-severe TBI developed NDD compared to 2.3% of persons with a history of mild TBI. In the Cox proportional hazards model, TBI was associated with an increased risk for NDD, with a HR of 1.8 (95% CI 1.6–2.1, *p <* 0.001), providing additional support for the results from the primary analysis.

A reviewer requested additional analysis to show how the risk of dementia behaved as a function of time (in the matched sample cohort). This analysis did not exclude persons diagnosed with dementia within the first year after the TBI. The risk for dementia was continually higher for persons with a history of moderate-to-severe TBI compared those with a history of mild TBI (S1 Fig). Furthermore, the lines diverge with time, providing further support for the association between moderate-to-severe TBI and dementia.

## Discussion

In this nationwide study in Finland investigating the association between TBI and NDD, we found that persons with a history of moderate-to-severe TBI had an 80% increased probability of future NDD compared to persons with a history of mild TBI. The matched sample analysis strengthened our results. The risk of future NDD increased with TBI severity (length of hospital stay) in a dose–response pattern. However, when the three NDD subtypes were analyzed separately, TBI was associated with increased risk only for dementia (90% increased probability).

Similar to our findings, three previous meta-analyses found TBI to be associated with an increased risk for dementia [3–5]. We further found that the associated risk between NDD and dementia increased with TBI severity. Thus, the evidence for the association between TBI and dementia seems to be compelling. Conversely, the association of TBI with PD and ALS is not as clear. Some studies suggest a significant association, and some no association [6,16–19]. In the present study, no association between moderate-to-severe TBI and PD or ALS could be established. The absolute numbers of persons developing PD and ALS were limited. Thus, the negative finding might be the consequence of a type II error. Yet, considering that this was an almost 30-year-long nationwide follow-up study including all persons hospitalized for TBI in Finland, ALS and PD do not seem to be a significant long-term neurological problem in individuals with a history of moderate-to-severe TBI. It should be noted that we were only able to identify persons diagnosed with ALS and PD who had been hospitalized. Thus, it is possible that we missed persons diagnosed with ALS or PD in the primary care setting or by private sector specialists.

In the present study, we could not study the risk of dementia in persons without a history of mild or moderate-to-severe TBI. However, based on previous studies, 14,500 persons are diagnosed with dementia annually in Finland. [20]. For a Finnish adult population (18 years or older) of 4,385,426, this translates to an incidence of 331 per 100,000 person-years. In comparison, the unadjusted incidence of dementia was 293 per 100,000 person-years for persons with a history of moderate-to-severe TBI and 114 per 100,000 person-years for persons with a history of mild TBI. Thus, working-aged persons with a moderate-to-severe injury at a young age have a similar incidence of dementia as the general population, where most cases are among elderly individuals [21]. The incidence of dementia in persons with a history of mild TBI is somewhat lower than the general incidence, most probably because our cohort included persons under 65 years of age at the time of injury.

A major limitation for comparing previous studies of the association between TBI and NDD is the large variation in TBI and NDD definitions [5]. Some studies rely upon self-reported diagnoses for TBI (and thus suffer from recall bias), while others use ICD diagnoses (eight, ninth, or tenth revisions). For NDD diagnosis, some studies use DSM criteria, ICD diagnoses, or NINCDS-ADRDA diagnostic criteria. Thus, not surprisingly, with such a wide spectrum of the basic definitions, the results have been conflicting. In this study, we defined mild TBI according to the CDC criteria [13]. The same definition was used by Gardner et al. [8], who showed that persons under the age of 65 years with mild TBI do not have an increased risk for dementia. Thus, as we specifically investigated persons under 65 years of age, the mild TBI population formed a suitable control group for individuals with moderate-to-severe TBI. Persons treated for mild and moderate-to-severe TBI are likely to be similar in TBI-specific risk factors, such as age, gender, alcohol use, and socioeconomic factors [22]. Yet, the included mild TBI population might differ somewhat from the most common form of mild TBI, when hospital admission is not required. Identifying a control group of individuals with a history mild TBI without hospitalization is, however, impossible in large epidemiological studies such as the present study.

The role of sex differences in risk of developing dementia after TBI has been widely discussed. Both Mortimer et al. [4] and Fleminger et al. [3] found men, but not women, with a history of TBI to have an increased risk of dementia after TBI. Speculated theories include estrogen- and progesterone-induced neuroprotection [23,24]. In our study, both men and women with a history of moderate-to-severe TBI had an increased risk for dementia compared to those with a history of mild TBI. Yet, female sex was associated with a reduced risk for dementia in comparison to male sex, both in the mild TBI and moderate-to-severe TBI groups. Population studies generally do not find differences in dementia incidence between men and women [25,26]. Our study cannot establish any causation between sex, TBI, and dementia, although our results imply that sex may play a role in TBI-related dementia. The underlying causes of TBI may be different in men and women, resulting in a differential capacity to recover from brain injury and hence differential risk of dementia.

Poor socioeconomic factors (such as low education level and socioeconomic group) increase the risk for sustaining a TBI [9]. However, they also serve as a risk factor future NDD [27]. Thus, the association between TBI and NDD in previous studies may have reflected the underlying association of these socioeconomic factors, rather than of TBI itself, with NDD. Yet, we found that even after adjusting for socioeconomic group and level of education, TBI was significantly associated with an increased risk for NDD.

### Strengths and limitations

This is, to our knowledge, the first nationwide study on the subject (including over 40,000 persons). The nationwide coverage and the high data quality of the registries strengthen the study’s generalizability [11,12]. Only a few previous studies match the present one in size. One of the larger studies (with a maximum follow-up time of 5 to 7 years) was by Gardner et al. [8], in which the authors identified approximately 50,000 persons with a history of TBI and found results like ours, i.e., moderate-to-severe TBI increased the risk for dementia, with a HR of 1.7 (compared to 1.9 in our study) [8]. Yet, to date, the present study has one of the longest follow-up times (mean time at risk 11 years, or 453,079 person-years), something that is essential when investigating long-term neurological morbidity after TBI. By using persons with a hospitalization due to mild TBI as controls, we diminished the likelihood of detecting an effect that is not present (i.e., type I error), as it is unlikely that persons with a history of mild TBI have an increased risk of dementia compared to persons with a history of non-brain trauma [8]. Yet, there are studies suggesting that mild TBI itself might increase the risk for NDD, and therefore it is possible that our results underestimate the effect of moderate-to-severe TBI in the development of NDD [28]. On the other hand, a recent systematic review found no association between mild TBI and dementia [29].

Despite the high quality of the registries used, all register-based studies include diagnostic inaccuracies, coding errors, and other confounding factors that cannot be controlled for. First, as the Care Register for Health Care includes only hospitalized persons; it is possible that we missed persons being diagnosed with NDD in the outpatient setting (e.g., milder forms of PD and dementia). Second, although the median follow-up time was 10 years, persons hospitalized for TBI during the more recent years inevitably had shorter follow-up times and may not yet have been diagnosed with a NDD. Third, a notably higher proportion of persons in the moderate-to-severe TBI group died during the follow-up period compared to the mild TBI group (29% versus 12%), decreasing the moderate-to-mild TBI cases’ exposure time. Thus, it is likely that the risk of NDD in persons with a history of moderate-to-severe TBI is even higher than presented in this study. Furthermore, the diagnosis of NDD, especially dementia, is prone to error if it occurs too soon after TBI. Such diagnosis may be a residual effect of delirium, medication, or other complications following TBI. To avoid the possibility of reverse causality, we recorded NDD diagnoses starting 1 year following the TBI. Yet, even after including persons diagnosed with dementia within the first year after the TBI, moderate-to-severe TBI was associated with a significantly higher risk for dementia than mild TBI, strengthening the association (S1 Fig).

As in many register-based studies, we used ICD-9 and ICD-10 discharge diagnoses to identify persons with a history of moderate-to-severe TBI and mild TBI [30]. For mild TBI we used diagnoses indicating no structural intracranial injury, and for moderate-to-severe TBI, we used diagnoses indicating an objective intracranial injury [31]. We further excluded persons with mild TBI hospitalized for longer than 1 day, as these may have had an undiagnosed intracranial injury, and persons with moderate-to-severe TBI hospitalized for shorter than 3 days, as these may represent cases of either rapid death or a milder form of intracranial injury not requiring hospitalization. It is possible that in the mild TBI group there were persons with clinically silent diffuse axonal injuries that passed undetected. How such traumatic microlesions affect the risk of future NDD is unknown.

The most evident limitation, which is shared by most large-scale epidemiological studies, is that the study setup does not allow to us analyze any causative factors. It has been hypothesized that TBI does not itself cause NDD but rather accelerates an underlying process of developing NDD in persons with predisposing factors [32]. Such predisposing factors may include comorbidities; genetic variations, such as APOE ε4 allele expression and neprilysin polymorphism; and lifestyle factors, such as cognitive reserve, physical activity, obesity, alcohol, and smoking [33]. For example, a substantial proportion of TBIs in Finland relate to alcohol use [34]. Alcohol-related TBIs are much more common in men than in women, especially among less educated people, who also have a higher baseline risk for NDD, which may confound our results [35,36]. Furthermore, comorbidities, such as hypertension [37], stroke [38], and diabetes [39], have been found to significantly associate with risk of NDD, especially with dementia. Therefore, differences in comorbidities between the mild and moderate-to-severe TBI groups may potentially have affected our results. The aspect of physical activity after TBI is interesting. Decreased physical activity is probably more likely to happen after moderate-to-severe TBI than after mild TBI. Decreased physical activity is associated with an increased risk for dementia [40,41]. Thus, increasing physical activity in persons after moderate-to-severe TBI, in combination with aggressive treatment of cardiovascular comorbidities, might decrease the risk of subsequent dementia. Yet, further studies investigating the causative relationship between TBI, other environmental risk factors, and genetics are needed.

### Conclusion

Our results suggest that in working-aged persons, moderate-to-severe TBI increases the risk for developing NDD later in life. In our study, the risk seemed to increase with TBI severity in a dose–response pattern, and the risk was higher in men. With regard to the NDD subtypes, moderate-to-severe TBI was associated with increased risk for dementia but not for PD and ALS. The effect of covariates, such as comorbidities, lifestyle factors, and genetic factors, should be accounted for in future etiological studies, as well as studies to improve diagnostics and prevention of dementia after TBI.

## Supporting information

S1 Fig Kaplan–Meier survival curves showing risk for dementia as a function of time in the matched sample cohort. Persons diagnosed with dementia within the first year after TBI were included as well. The risk for dementia was continually higher in the moderate-to-severe TBI group than in the mild TBI group. Further, the lines diverge with time, strengthening the association between moderate-to-severe TBI and dementia.
(TIF)

S1 RECORD Checklist The REporting of studies Conducted using Observational Routinely-collected Data (RECORD) checklist. (DOCX)

S1 Table Baseline differences between persons with a history of moderate-to-severe traumatic brain injury and persons with a history of mild traumatic brain injury who went on to develop neurodegenerative disease. (DOCX)

S2 Table Baseline matching characteristics in 1:1 matched persons with a history of moderate-to-severe traumatic brain injury and mild traumatic brain injury. (DOCX)

S1 Text List of socioeconomic groups and education levels. (DOCX)

S2 Text Preplanned analysis plan. (DOCX)

## Abbreviations

ALS: amyotrophic lateral sclerosis
CDC: US Centers for Disease Control and Prevention
HR: hazard ratio
NDD: neurodegenerative disease
PD: Parkinson disease
TBI: traumatic brain injury
