# Supplementary figures and images for: Risk of hospitalization with neurodegenerative disease after moderate-to-severe traumatic brain injury in the working-age population: A retrospective cohort study using the Finnish national health registries
Source: PLoS Med. 2017 Jul 5;14(7):e1002316. doi: 10.1371/journal.pmed.1002316 (PMC5497945; doi:10.1371/journal.pmed.1002316)

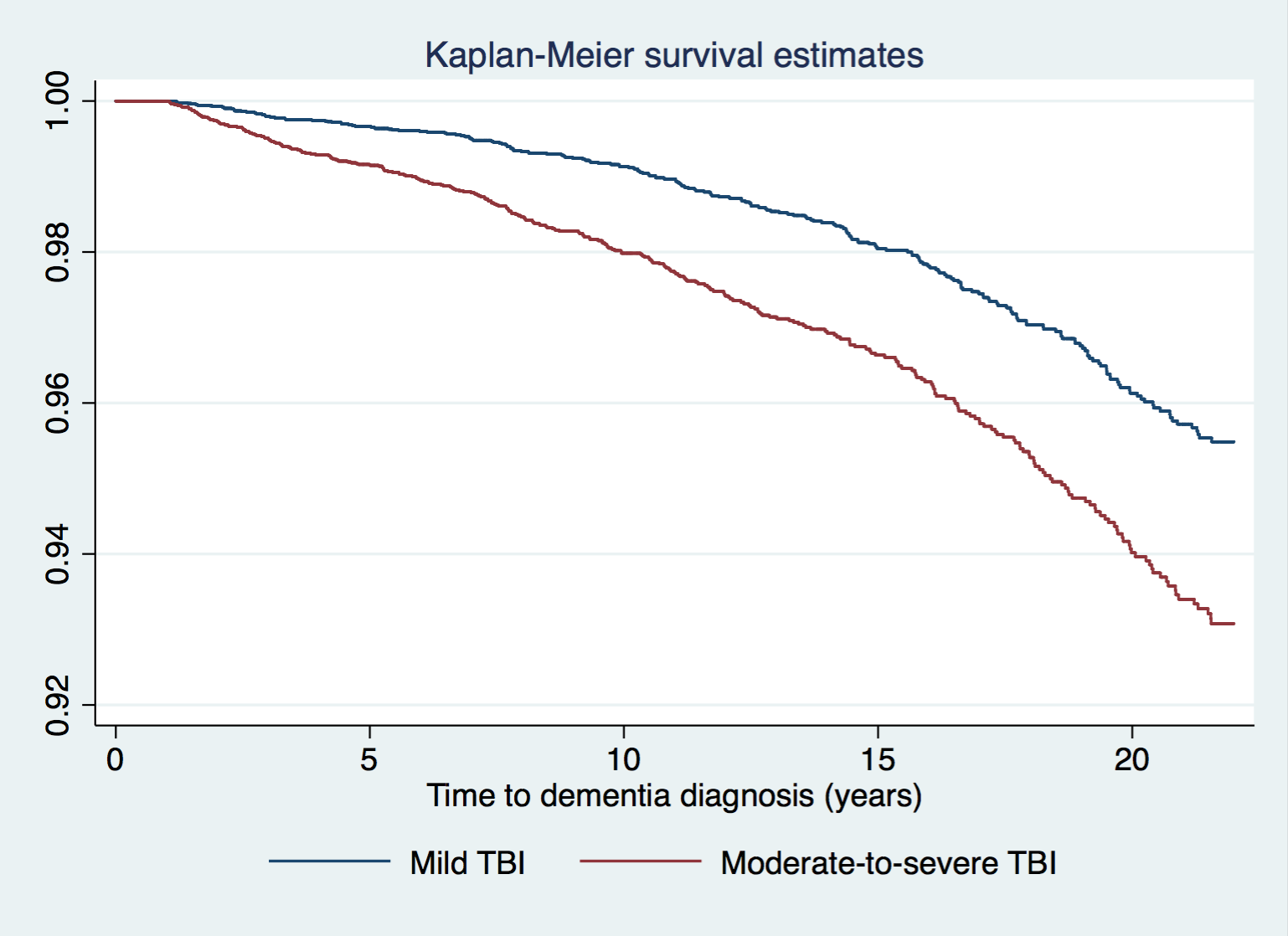

Supplement: S1 Fig — Persons diagnosed with dementia within the first year after TBI were included as well. The risk for dementia was continually higher in the moderate-to-severe TBI group than in the mild TBI group. Further, the lines diverge with time, strengthening the association between moderate-to-severe TBI and dementia. (TIF) [file pmed.1002316.s001.tif]
