# Supplementary material for: Risk of hospitalization with neurodegenerative disease after moderate-to-severe traumatic brain injury in the working-age population: A retrospective cohort study using the Finnish national health registries
Source: PLoS Med. 2017 Jul 5;14(7):e1002316. doi: 10.1371/journal.pmed.1002316 (PMC5497945; doi:10.1371/journal.pmed.1002316)
Supplement: S1 Table — (DOCX) [file pmed.1002316.s003.docx]

| **S1 Table:** Baseline differences between persons with a history of moderate-to-severe traumatic brain injury or mild TBI who went on to develop neurodegenerative disease. | | | |
| --- | --- | --- | --- |
|  | **Persons developing NDD** (N=1,022) | | |
| **Variable** | **Moderate-to-severe TBI**  (N=696) | **Mild TBI**  (N=326) | **p-Value*** |
| **Age at injury** |  |  |  |
| Mean (SD) | 56 (8) | 58 (8) | 0.016 |
| 18-40 years | 36 (5%) | 13 (4%) | 0.107 |
| 41-50 years | 91 (13%) | 30 (9%) |  |
| 51-60 years | 310 (45%) | 140 (43%) |  |
| 61-65 years | 259 (37%) | 143 (44%) |  |
| **Sex** |  |  |  |
| Male | 508 (73%) | 167 (51%) | <0.001 |
| Female | 188 (27%) | 159 (49%) |  |
| **Socio-economic group** |  |  |  |
| Self-employed | 1 (0%) | 0 (0%) | 0.174 |
| Upper-level employees | 1 (0%) | 1 (0%) |  |
| Lower-level employees | 2 (0%) | 0 (0%) |  |
| Manual workers | 1 (0%) | 3 (1%) |  |
| Students | 0 (0%) | 0 (0%) |  |
| Pensioners | 678 (98%) | 319 (98%) |  |
| Unemployed | 5 (1%) | 3 (1%) |  |
| Unknown | 8 (1%) | 0 (0%) |  |
| **Education level** |  |  |  |
| Upper-secondary | 176 (25%) | 68 (21%) | 0.459 |
| Short-cycle tertiary | 47 (7%) | 17 (5%) |  |
| Bachelor or equivalent | 28 (4%) | 11 (3%) |  |
| Master or equivalent | 14 (2%) | 9 (3%) |  |
| Doctor or equivalent | 2 (0) | 1 (0%) |  |
| Unknown | 429 (62%) | 220 (68%) |  |
| **Hospital length of stay**† |  |  |  |
| 3-5 days | 118 (17%) | NA | NA |
| 6-10 days | 183 (26%) | NA |  |
| 11-24 days | 207 (30%) | NA |  |
| ≥25 days | 188 (27%) | NA |  |
| **NDD type** |  |  |  |
| Dementia | 615 (88%) | 276 (84%) | 0.245 |
| PD | 68 (10%) | 41 (13%) |  |
| ALS | 13 (2%) | 9 (3%) |  |
| **Age at NDD** |  |  |  |
| Mean (SD) | 67 (19) | 71 (10) | <0.001 |
| ≤65 years | 275 (40%) | 84 (26%) | <0.001 |
| >65 years | 421 (60%) | 242 (74%) | <0.001 |
| **Mean time to NDD**, years | 10 (6) | 14 (7) | <0.001 |
| *Categorical variables are tested using a two-sided χ^2^-test and continuous variables tested using a t-test. The reported p-values are not age and sex adjusted.  †Hospital length of stay due to the TBI. All persons in the mild TBI group had a length of stay of 0 to 1 day  *Abbreviations*: ALS, Amyotrophic Lateral Sclerosis; IQR, interquartile range; NDD, neurodegenerative disease; LOS, length of stay; SD, standard deviation; TBI, traumatic brain injury; PD, Parkinson’s disease. | | | |
