# Supplementary material for: Risk of hospitalization with neurodegenerative disease after moderate-to-severe traumatic brain injury in the working-age population: A retrospective cohort study using the Finnish national health registries
Source: PLoS Med. 2017 Jul 5;14(7):e1002316. doi: 10.1371/journal.pmed.1002316 (PMC5497945; doi:10.1371/journal.pmed.1002316)
Supplement: S2 Table — (DOCX) [file pmed.1002316.s004.docx]

| **S2 Table**: Baseline matching characteristics in 1:1 matched persons with a history of moderate-to-severe TBI and mild TBI | | |
| --- | --- | --- |
|  | **Moderate-to-severe TBI (N=12,227)** | **Mild TBI (N=13,470)** |
| **Age** | 44.1 (13.8) | 44.3 (13.7) |
| **Female** | 3,938 (32%) | 4,235 (31%) |
| **Socio-economic group** |  |  |
| Self-employed | 415 (3%) | 487 (4%) |
| Upper-level employees | 456 (4%) | 508 (4%) |
| Lower-level employees | 884 (7%) | 985 (7%) |
| Manual workers | 1,499 (12%) | 1,742 (13%) |
| Students | 220 (2%) | 262 (2%) |
| Pensioners | 6,868 (56%) | 7,171 (53%) |
| Unemployed | 1,451 (12%) | 1,684 (13%) |
| Unknown | 484 (4%) | 631 (4%) |
| **Level of education** |  |  |
| Upper secondary | 5,448 (45%) | 5,977 (45%) |
| Short-cycle tertiary | 942 (8%) | 1,007 (8%) |
| Bachelor or equivalent | 535 (4%) | 567 (4%) |
| Master or equivalent | 426 (3%) | 454 (3%) |
| Doctor or equivalent | 23 (0%) | 23 (0%) |
| Unknown | 4,903 (40%) | 5,442 (40%) |
| Age showed as mean (standard deviation), female, level of education, and socio-economic group showed as N (percentage) | | |
