# Supplementary material for: Risk of hospitalization with neurodegenerative disease after moderate-to-severe traumatic brain injury in the working-age population: A retrospective cohort study using the Finnish national health registries
Source: PLoS Med. 2017 Jul 5;14(7):e1002316. doi: 10.1371/journal.pmed.1002316 (PMC5497945; doi:10.1371/journal.pmed.1002316)
Supplement: S1 Text — (DOCX) [file pmed.1002316.s005.docx]

# **S1 Text: Socio-economic groups and education levels**

## **Socio-economic groups**

- Self-employed persons
- Upper-level employees with administrative, managerial, professional and related occupations
- Lower-level employees with administrative and clerical occupations
- Manual workers
- Students
- Pensioners

## **Levels of education**

- General education
- Primary education
- Lower secondary education
- Upper secondary education
- Post-secondary non-tertiary education
- Short-cycle tertiary education
- Bachelor´s or equivalent education
- Master´s or equivalent education
- Doctoral or equivalent education
