# Supplementary material for: Risk of hospitalization with neurodegenerative disease after moderate-to-severe traumatic brain injury in the working-age population: A retrospective cohort study using the Finnish national health registries
Source: PLoS Med. 2017 Jul 5;14(7):e1002316. doi: 10.1371/journal.pmed.1002316 (PMC5497945; doi:10.1371/journal.pmed.1002316)
Supplement: S2 Text — (DOCX) [file pmed.1002316.s006.docx]

**S2 Text: ANALYSIS PLAN**

**AIM**

The aim is to assess the association between neurodegenerative disease (NDD) and moderate-to-severe traumatic brain injury (TBI) on a population level by comparing persons hospitalized due to moderate-to-severe TBI (diagnosis indicating structural intracranial injury) and persons hospitalized due to mild TBI (diagnosis not indicating structural intracranial injury).

**METHODS**

*Study Population*

Moderate-to-severe TBI is defined as an ICD-9 or ICD-10 diagnosis code indicating structural intracranial injury (Table 1). To diminish the likelihood of persons with moderate-to-severe TBI being misdiagnosed we will only include persons hospitalized for 3 days or longer. As we are limited to the data found in the National Health Registry, we will hospital length of stay (LOS) as a surrogate markers of injury severity (longer LOS indicating a more severe injury).

Mild TBI is defined as an ICD-9 or ICD-10 code indicating head trauma without any structural intracranial injury (Table 1). To diminish the likelihood of persons with mild TBI having a more severe injury we will only include persons being hospitalized for a maximum of one day.

We will include persons between 18 to 65 years of age (working-age) and admitted between 1987 and 2014 (ICD-9 and ICD-10 were used).

| **Table 1:** Traumatic brain injury related diagnoses according to the ICD-9 and ICD-10 | | |
| --- | --- | --- |
|  | **Mild TBI***  **(no structural intracranial injury)** | **Moderate-to-severe TBI†**  **(structural intracranial injury)** |
| **ICD-9** | 850 | 851, 852, 853, 854 |
| **ICD-10** | S06.0 | S06.1, S06.2, S06.3, S06.4, S06.5, S06.6, S06.7, S06.8, S06.9 |
| ICD, International Statistical Classification of Diseases and Health Related Problems  *Hospitalized for a maximum of one day  †Hospitalized for a minimum of three days | | |

Neurodegenerative disease was defined according to ICD-9 and ICD-10 diagnoses (Table 2). Time of hospitalization due to the NDD was considered time of diagnosis. To avoid the possibility of reverse causality we excluded persons receiving the NDD diagnosis within one-year of the TBI. We further excluded persons dying within one-year of injury, as NDD is a long-term neurological problem progressing over years.

| **Table 2:** Neurodegenerative disease related diagnoses according to the ICD-9 and ICD-10 | | | |
| --- | --- | --- | --- |
|  | **Parkinson’s disease** | **Dementia** | **Amyotrophic Lateral Sclerosis** |
| **ICD-9** | 332 | 290, 331, 797 | 335.2 |
| **ICD-10** | G20 | G30, F00, F02, F01, F03 | G12.2 |
| Abbreviations: ICD, International Statistical Classification of Diseases and Health Related Problems | | | |

*Date of death, follow-up and socioeconomic status*

Data on mortality will be obtained through the Finnish Population Register Center. Persons emigrating from Finland pose a potential risk for loss of follow-up. For these persons, their last known date of living in Finland will serve as their end of follow-up. For all other persons, the end of follow-up will be regarded as 1) date of death or 2) end of 2014. Person education and socioeconomic status will be obtained through the Statistics Central of Finland. The registries are linked by the patients´ individual unique social security numbers.

*Analyses*

Stata (version 14, StataCorp, College Station, TX) was used for the statistical analyses.

Univariate analysis is performed using a two-sided chi-squared test (categorical) and a Student´s t-test (normally distributed continuous data).

In the primary analysis, we will assess the association between moderate-to-severe TBI and NDD, using the mild TBI group as the reference. For this, we will use a Cox proportional hazards model adjusting for gender, socio-economic group and level of education. Age is the underlying parameter in the Cox model and, thus, automatically adjusted for.

In the sensitivity analyses we will separately assess the association between moderate-to-severe TBI and dementia, Parkinson´s disease (PD) or Amyotrophic Lateral Sclerosis (ALS) by using the above-mentioned Cox model, adjusting for the same predictors.

In the subgroup analyses we will assess the association between moderate-to-severe TBI and NDD by gender, by pre-specified age groups (18 to 40 years, 41 to 50 years, 51 to 60 years, and 61 to 65 years) and by hospital LOS quartiles. Hospital LOS is a surrogate marker of injury severity.

To validate our results, we will perform a matched sample analysis. We will match persons with a history of moderate-to-severe TBI and mild TBI in a 1:1 fashion based on age, sex and socioeconomic group. In the matched sample a simple Cox model will be used to assess the association between moderate-to-severe TBI and NDD.

The results will be presented as hazards ratios (HR) with 95% confidence intervals (CI). P-values <0.05 will be considered statistically significant.
